# Squamous Cell Carcinoma of the Thumb: A Case Report

**DOI:** 10.7759/cureus.54046

**Published:** 2024-02-12

**Authors:** Georgi P Georgiev, Julian M Ananiev, Violeta Groudeva

**Affiliations:** 1 Department of Orthopedics and Traumatology, University Hospital Queen Giovanna, Medical University of Sofia, Sofia, BGR; 2 Department of General and Clinical Pathology, Forensic Medicine, Deontology and Dermatovenerology, Trakia University, Stara Zagora, BGR; 3 Department of Diagnostic Imaging, University Hospital St. Ekaterina, Medical University of Sofia, Sofia, BGR

**Keywords:** hand, treatment, diagnosis, thumb, squamous cell carcinoma

## Abstract

Squamous cell carcinoma (SCC) is the most common tumor of the hand with a high tendency for local recurrence and a low rate of metastasis. Herein, we present an interesting case of SCC of the thumb of the right hand in a 68-year-old patient with one recurrence, treated with surgical excision and following radiotherapy. Five years postoperative, there are no clinical and imaging data for local recurrence, as well as the presence of metastases. We also make a brief review of the current literature on this neoplasia.

## Introduction

Squamous cell carcinoma (SCC) is the most common malignant skin tumor on the hand [1]. Fifteen percent of cases are localized in the hand area, with the dorsal surface being the most common location, followed by the back of the fingers, the web space, and the skin on the dorsal side of the wrist [1,2]. SCC originates from the stratum spinosum of the epithelium and is predominantly observed in older men. This tumor initially presents as nodules, which later develop areas of necrosis. Its macroscopic appearance varies from small, dry, scaly erythematous lesions to large, rapidly growing masses with a fungating shape. SCC usually arises in precancerous formations after significant sunburns, actinic keratosis, leukoplakia, radiation keratosis, scars, and chronic ulcers [3].

There are generally two types of SCC observed. The slow-growing verrucous type is often exophytic with a high tendency for metastasis, while the second type is nodular and indurated with rapid growth and early ulceration. Highly differentiated SCCs have a recurrence rate of 7%, whereas poorly differentiated tumors show recurrence in 28% of cases [4]. This neoplasm has a high metastatic potential, with the risk of metastasis in lesions affecting the extremities ranging from 2% to 5% [1,5]. Tumors with a diameter over 2 cm are twice as likely to recur (15.2% vs. 7.4%) and three times more likely to metastasize (30.3% vs. 9.1%) compared to smaller tumors [6].

The purpose of this article is to present a case of SCC located on the thumb of a 68-year-old patient, which was surgically treated on two occasions followed by postoperative radiotherapy.

## Case presentation

**Figure 1:**
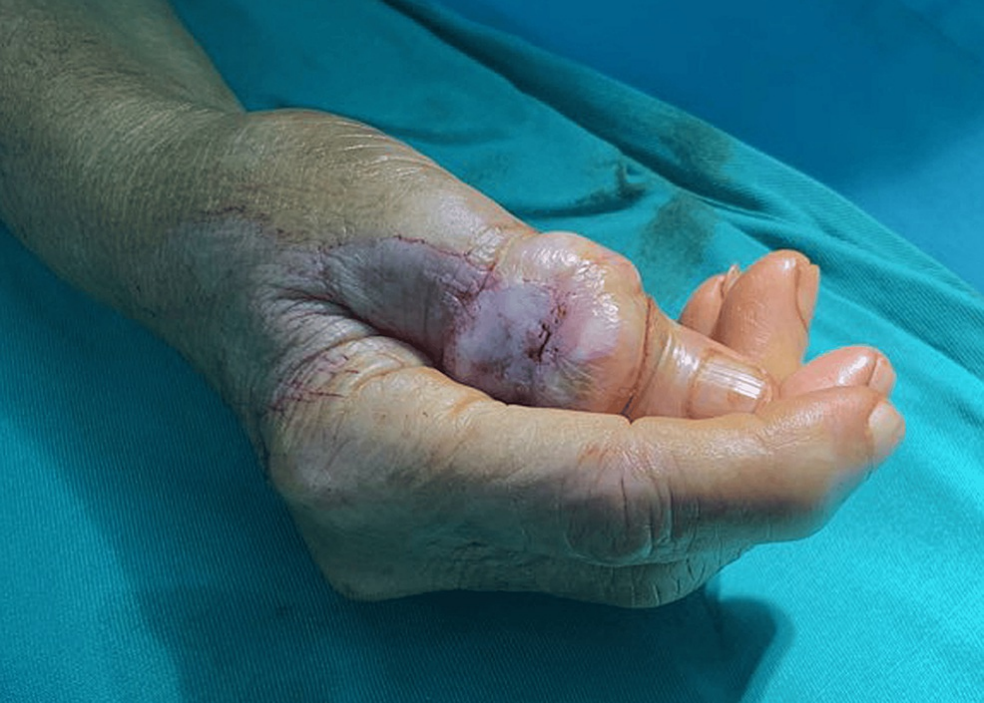
Preoperative photography of ulcerated lesion on the dorsal surface of the thumb involving the skin more than half of the circumference of the thumb

A 68-year-old female patient presented for examination at the clinic with a painless, ulcerated lesion on the dorsal surface of the thumb involving the skin (more than half of the circumference of the thumb). The formation appeared approximately three months ago and rapidly grew. There were no historical data of trauma in the area. Upon clinical examination, the dorsal surface of the thumb in the interphalangeal joint area was affected by a well-defined formation with a firm consistency and slight mobility, measuring 28 mm in diameter (Figure 1).

**Figure 2:**
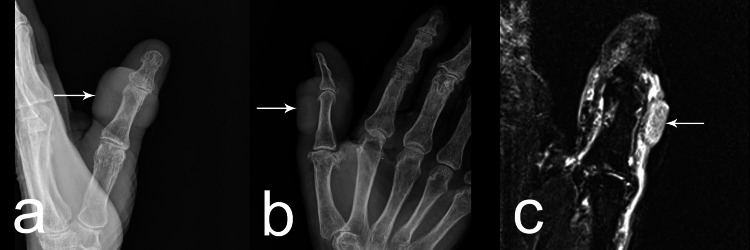
a,b: Roentgenography presented a clearly defined radiolucent ovoid formation in the thumb area (arrow) without involvement of bone structures (a – lateral view; b - anterior posterior view); c - Magnetic resonance imaging presented a semi-sleeve-shaped soft tissue heterogeneous formation (arrow) covering the dorso-lateral part of the finger's circumference.

Roentgenography revealed a clearly defined radiolucent ovoid formation in the thumb area without involvement of bone structures (Figure 2a, 2b). Magnetic resonance imaging (MRI) showed a semi-sleeve-shaped soft tissue heterogeneous formation covering the dorsolateral part of the finger's circumference (Figure 2c).

**Figure 3:**
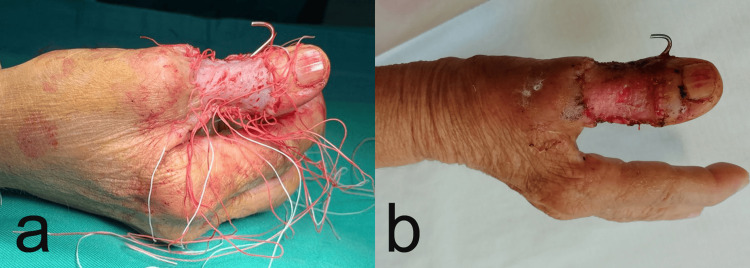
a) Intraoperative photography presenting the defect after excision covered with a split-thickness skin graft taken from the volar side of the distal forearm.; b) graft after suture removal and immobilized with a K-wire interphalangeal joint after 2 weeks.

Surgical treatment was performed through circumferential excision of the tumor with a 5 mm clear margin, and the resulting defect was covered with a split-thickness skin graft taken from the volar side of the distal forearm (Figures 3a). The interphalangeal joint of the thumb was protected and immobilized with a K-wire for two weeks (Figure 3b). 

**Figure 4:**
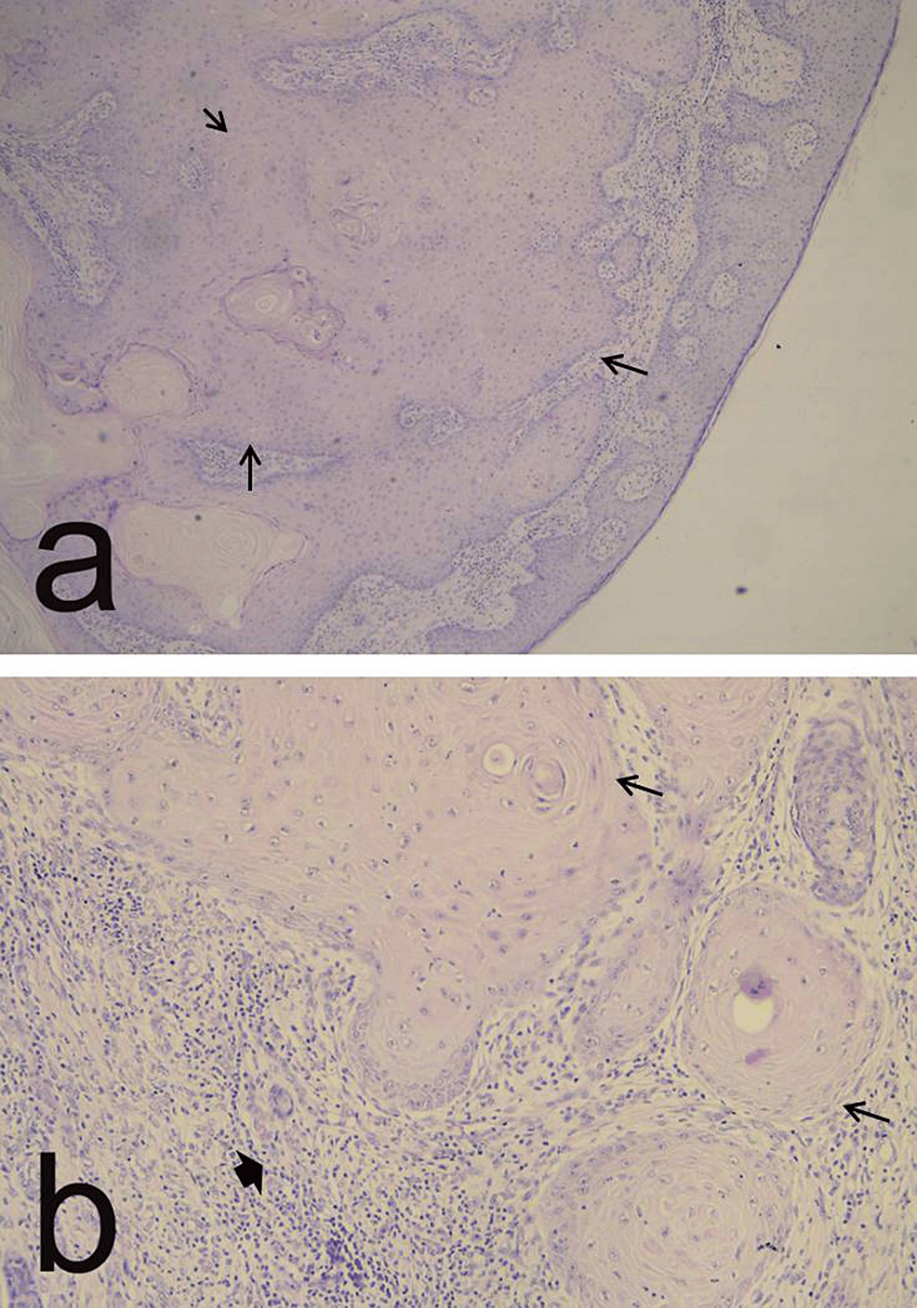
Microscopic appearance: a) well-differentiated squamous cell carcinoma (G1) with keratinization, intercellular bridges and minimal pleomorphism and mitoses (arrows) (magnification x 100); b) well-differentiated squamous cell carcinoma with keratin pearls (arrows) surrounded by an desmoplastic stromal reaction (arrowhead) (magnification x 200).

The histological diagnosis revealed a well-differentiated squamous cell carcinoma G1 with keratinization (Figure 4a, 4b) and clear resection margins. 

**Figure 5:**
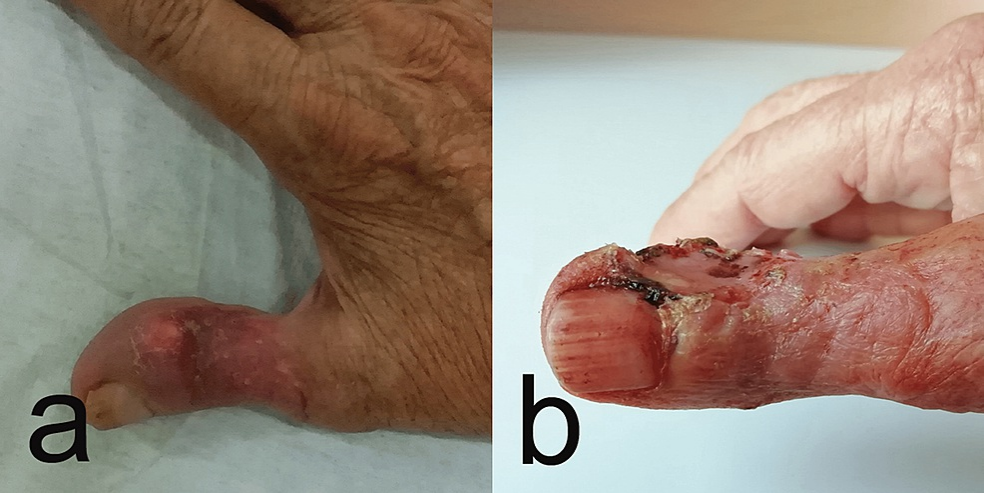
a) photograph showing the recurrence on the dorso-ulnar side of the thumb; b) Postoperative photography after suture removal: after 2 weeks, the patient presented the covered defect with a split-thickness skin graft after excision.

**Figure 6:**
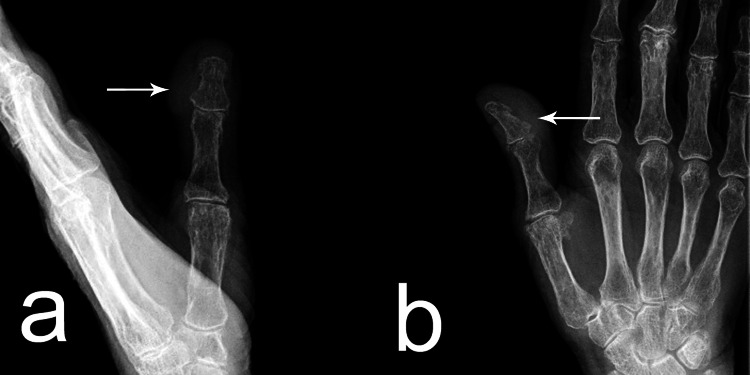
Roentgenography presented a clearly defined soft tissue formation in the thumb area without involvement of bone structures (a – anterior-posterior view; b - lateral view).

The postoperative period proceeded without complications. After the removal of the K-wire on day 14, the patient was encouraged to start using the thumb in her daily activities. No specific rehabilitation protocol was used. At the end of the first month, the patient started weight-bearing with hand and a normal return to daily activities. After the first month, a course of radiotherapy was administered. However, at the three-month follow-up, a recurrence was identified on the dorso-ulnar aspect of the thumb (Figure 5a). Subsequent X-ray examinations revealed no involvement of the bone structures (Figure 6). To address this recurrence, a repeat circumferential excision was performed with a 5 mm clear margin following oncological principles, and the resulting defect was reconstructed using a split-thickness skin graft (Figure 5b).

**Figure 7:**
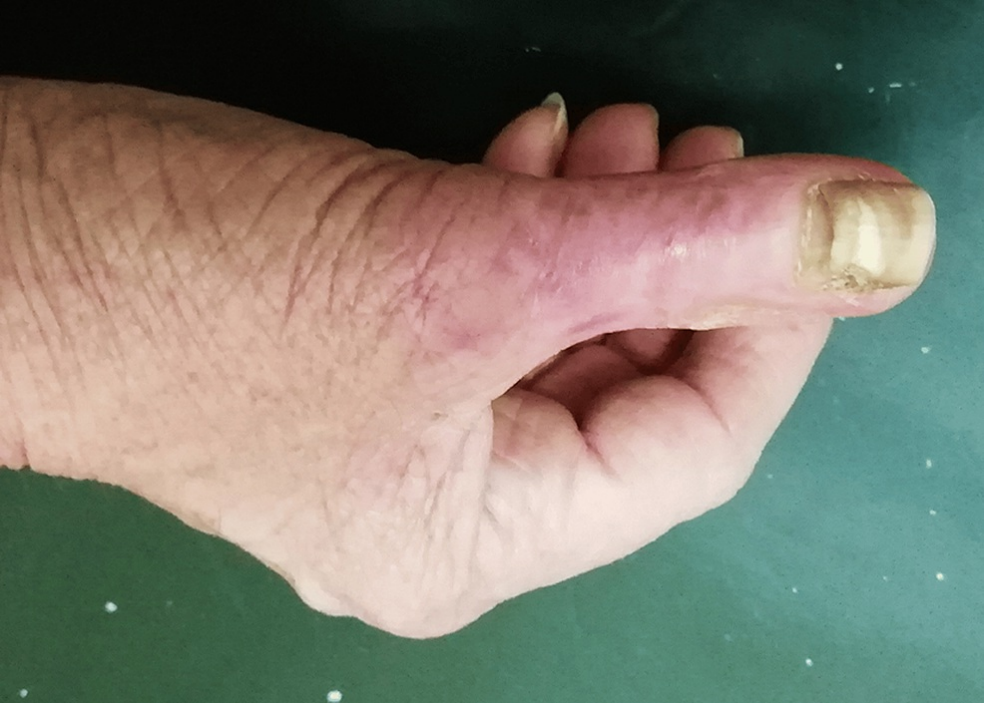
Postoperative photography: fifth year after removal

Histopathological analysis confirmed the initial diagnosis. The postoperative period was uneventful. Again, no specific rehabilitation protocol was used. At the end of the first month, the patient returned to her daily activities and full weight-bearing of the hand. Upon nearly five years of follow-up, there has been no evidence of local recurrence or metastasis (Figure 7).

## Discussion

Tumors of the hand represent 10 to 15% of all malignant skin and soft tissue diseases [7]. SCC accounts for approximately 20% of all skin carcinomas and nearly 75% of all malignant skin lesions in the hand region [8,9]. Risk factors include fair skin, cumulative excessive exposure to ultraviolet radiation, advanced age, and chronically damaged skin. Additional hereditary factors include xeroderma pigmentosum and albinism [1].

Histologically, all SCCs share similar characteristics. These tumors consist of irregular nests and sheets of flattened epithelium extending into the dermis. The cellular differentiation and the number of atypical pleomorphic cells determine the degree of tumor differentiation. Well or highly-differentiated tumors exhibit keratin pearls surrounded by epithelial cells and individual keratinizing cells. Cellular differentiation and keratinization are significantly reduced in poorly differentiated tumors, while mitoses increase [1,5]. As with other neoplasms, computed tomography (CT) and MRI are crucial for evaluating SCC [10-12]. CT visualizes cortical erosion, the degree of bone involvement, and the presence of pathological fractures. MRI surpasses other imaging methods for soft tissue tumors with the ability to identify the precise location and detect invasion into surrounding structures [10].

Treatment of SCC includes surgical excision, local destructive therapy, chemotherapy, and radiation therapy, with the primary choice being surgical intervention. Factors influencing treatment include tumor size, location, degree of invasion, and overall health. The risk of metastasis and the recurrence rate for SCC localized on the back of the hand is higher than in other locations [1,13-15]. In the presented case, to prevent the recurrence of an SCC during the first excision of the tumour, a larger clear surgical excision with a margin more than 5 mm needs to be performed.

The hand surgeon has two main goals: the excision of the tumor with a good oncological outcome and the aesthetic and functional reconstruction of the respective area [11]. SCCs are more aggressive than basal cell carcinomas for local control of the tumor formation, and a wider excision is necessary. Surgical margin recommendations vary from 0.5-1.0 cm for lesions under 3 cm to 1 cm for lesions over 3 cm [1,14,16]. Good oncological results after SCC surgery reach up to 94% [17]. Skin coverage after excision is achieved through primary closure or skin grafting. Amputation is indicated in cases of bone invasion [11]. Complications of surgical treatment may include the local formation of seroma, hematoma, or infection [11].

5-fluorouracil is also considered in the management. Radiation therapy is indicated for inoperable patients or those refusing surgical intervention, as well as postoperatively. Good oncological results reach up to 90% [18]. Drawbacks include prolonged treatment intervals, dermatitis, and fibrosis [1]. Destructive techniques, including electrodesiccation, curettage, and cryosurgery, are limited to small superficial lesions [1].

The recurrence rate after surgical treatment varies [1]. For lesions smaller than 2 cm, a local recurrence is observed in 7.4% and metastasis in 9.1%. In comparison, for tumors with a diameter over 2 cm, local recurrence is seen in 15.2% of cases and metastasis in 30.3% [18]. The most common sites of metastasis are regional epitrochlear and axillary lymph nodes. A 5-year survival rate of 39% is observed for metastases from hand SCC. Routine prophylactic lymph node dissection in the upper limbs for SCC is generally not indicated [1].

## Conclusions

Due to its role in hand function, surgical treatment of tumors on the thumb poses a real challenge for the hand surgeon. The primary goal is to achieve a good oncological outcome while preserving grip function. SCC occurring on the hand has a worse course, high recurrence rates, and could give metastasis. In some cases, a significant functional deficiency after excision, likely involving reconstruction surgery, could be established. A thumb amputation could be also used in the treatment algorithm. Thumb-preserving wide excision, as in the reported case, with good oncologic and acceptable functional outcomes is the ideal option. The preoperative assessment in these cases is crucial for successful clinical outcomes and the preservation of hand function.

## References

[1] TerKonda SP, Perdikis G (2004). Non-melanotic skin tumors of the upper extremity. Hand Clin.

[2] Wollina U, Tempel S, Albert W, Hansel G, Heinig B (2019). Advanced Ulcerated Squamous Cell Carcinoma of the Hand with Locoregional Axillary Lymph Node Metastasis - Case Report and Literature Review. Open Access Maced J Med Sci.

[3] Brownstein MH, Rabinowitz AD (1979). The precursors of cutaneous squamous cell carcinoma. Int J Dermatol.

[4] Immerman SC, Scanlon EF, Christ M, Knox KL (1983). Recurrent squamous cell carcinoma of the skin. Cancer.

[5] Møller R, Reymann F, Hou-Jensen K (1979). Metastases in dermatological patients with squamous cell carcinoma. Arch Dermatol.

[6] Clayman GL, Lee JJ, Holsinger FC (2005). Mortality risk from squamous cell skin cancer. J Clin Oncol.

[7] Martinez JC, Otley CC (2001). The management of melanoma and nonmelanoma skin cancer: a review for the primary care physician. Mayo Clin Proc.

[8] Bean DJ, Rees RS, O'Leary JP, Lynch JB (1984). Carcinoma of the hand: a 20-year experience. South Med J.

[9] Chakrabarti I, Watson JD, Dorrance H (1993). Skin tumours of the hand. A 10-year review. J Hand Surg Br.

[10] Inkaya E, Sayit E, Sayit AT, Zan E, Bakirtas M (2015). Subungual Squamous Cell Carcinoma of the Third Finger with Radiologic and Histopathologic Findings: A Report of Case. J Hand Microsurg.

[11] Irmak F, Şirvan SS, Serin M, Sevim KZ, Yeşilada AK, Yazar SK (2019). Squamous cell carcinoma of the hand: clinical presentation, surgical treatment, outcome and survival rate: a series of 129 cases. Turk J Dermatol.

[12] Maciburko SJ, Townley WA, Hollowood K, Giele HP (2012). Skin cancers of the hand: a series of 541 malignancies. Plast Reconstr Surg.

[13] Labow BI, Rosen H, Greene AK, Lee WP, Upton J (2008). Soft tissue sarcomas of the hand: functional reconstruction and outcome analysis. Hand (N Y).

[14] Motley R, Kersey P, Lawrence C (2002). Multiprofessional guidelines for the management of the patient with primary cutaneous squamous cell carcinoma. Br J Dermatol.

[15] Rayner CR (1981). The results of treatment of two hundred and seventy-three carcinomas of the hand. Hand.

[16] Luce EA (1995). Oncologic considerations in nonmelanotic skin cancer. Clin Plast Surg.

[17] Mohs FE (1980). Chemosurgery. Clin Plast Surg.

[18] Rowe DE, Carroll RJ, Day CL Jr (1992). Prognostic factors for local recurrence, metastasis, and survival rates in squamous cell carcinoma of the skin, ear, and lip. Implications for treatment modality selection. J Am Acad Dermatol.

